# Abstract of the Proceedings of the Buffalo Medical Association

**Published:** 1859-05

**Authors:** Austin Flint


ART. III.—*Abstract of Proceedings of the Buffalo Medical Association.*

Tuesday Evening, April 5, 1859.

The Association met.

Present—The President, Dr. Wyckoff, in the chair; and Drs. Hutchins, Rogers, Newman, Wilcox, Rankin, Gay, Butler, Miner, Congar, Strong, Lemon, Nichell, Hawley, and Flint, Jr.

The minutes of the last meeting were read and approved.

Dr. Hawley read the following report of a case of *Pseudo-membranous Croup:*

The following case of pseudo-membranous croup has no interest in itself more than any other rapid and fatal case of the same disease, except that it gave me an opportunity of successfully tubing and injecting the larynx.

The patient was a strong male infant, æt. 9 months. His symptoms had excited the alarm of the parents more than twenty-four hours before I saw him. The little sufferer breathed with the greatest conceivable difficulty; its face, tumid and livid, and the velum palati and fauces presented apthous spots.

The treatment consisted in the use of small doses of calomel frequently repeated, and attempts to apply nitrate of silver locally. I made repeated efforts, and believe some of them were successful. No immediate relief followed, but during the night the urgency of the symptoms abated materially. The introduction of the probang was again attempted, but I think, unsuccessfully. I then tried the introduction of a catheter, in which I succeeded, and injected a strong solution of nitrate of silver. The entrance of the tube was indicated by the passage of air through it.

Neither the entrance of the tube nor the injection of the fluid, increased the strangulation which the finger had caused. The tube, when withdrawn, was found filled at its end with dense whitish substance, too firm for mucus and too fluid for my idea of fibrinous deposit.

The child died about sixty hours after its seizure.

The association then adjourned till Tuesday evening following, deferring the report of the officers and the election ef officers, which was the order of business for the evening, until that time.

TUESDAY EVENING, April 12th, 1859.

Adjourned meeting.

Present—The President, Dr. Wyckoff, in the chair; and Drs. Newman, Flint, Miner, White, Rankin, Ring, Nichell, Butler, Hutchins, Rochester, C. L. Dayton, Whitney. Eastman, Wilcox, Hunt, Gay, and Flint, Jr.

The officers of the association then made their annual report.

The association then proceeded to the election of officers, and the following gentlemen were declared duly elected for the ensuing year:

| | |
|---|---|
| *President*, . . . | JAMES M. NEWMAN, M. D. |
| *Vice-President*, . . . | THOMAS F. ROCHESTER, M. D. |
| *Secretary*, . . . | AUSTIN FLINT, Jr., M. D. |
| *Treasurer*, . . . | C. B. HUTCHINS, M. D. |
| *Librarian*, . . . | BENJAMIN H. LEMON, M. D. |

Dr. FLINT, Jr., moved that a committee of three be appointed to conduct the newly elected president to the chair. Seconded and carried.

The chair appointed Drs. Wilcox, Hunt, and Whitney, such committee.

Dr. Newman then took the cnair with a few appropriate remarks.

The retiring president then read the following address:

*Gentlemen of the Association:*

In compliance with one of our by-laws, it becomes the duty of the retiring president to deliver an address upon some surgical or medical subject; I will invite your attention, therefore, to a few observations upon Chorea Sancti Viti, or vulgarly, Saint Vitus' Dance.

From the history of this disease, I conclude it has been more frequent the last few years than formerly. Although often confounded, and sometimes complicated with epilepsy, hysteria, catalepsy, and some forms of paralysis, yet it may generally be accurately diagnosed, notwithstanding the contrariety of symptoms in different cases. It is to this latter circumstance only that we can attribute the defective definitions found in nosological writers, none of whom have been able to include all the variety of phases it assumes, or even to give us one symptom that is uniform or pathognomonic.

From the variety of cases which have come under my own observation, differing so widely from each other in their symptomatology, I have come to the conclusion that a true portraiture of this malady, exhibiting signs that are uniformly present, is impracticable. One symptom, however, which is described by Dr. Todd, I have found more generally present than any other, and also, more peculiar and characteristic. That is the protrusion of the tongue with a thrust to the fullest extent of which it will admit; frequently this is done by one effort, at other times, it requires two or three attempts before it can be accomplished. And the subsequent retraction is also peculiar: the tongue is drawn back, supported and guided by the pressure of the teeth, and often very slowly and cautiously. In many cases, this is the first symptom of an approaching attack of chorea. Usually we find in the commencement of this disease, an occasional twitching of some of the muscles of the face, neck, mouth, or extremities. One of the most prominent symptoms, however, is a limping in the gait, occasioned by dragging one foot after the other, this is soon followed by an involuntary motion of one or both arms, which soon extends to the muscles of the lower extremities, and the face and neck, exhibiting in the countenance, ludricous and often hideous grimaces, accompanied by rapid and violent motions of the head and even sometimes of the entire body. These involuntary motions are sometimes periodical, and sometimes constant, continuing for weeks, except during sleep; and in some severe cases they are manifested even in sleep itself. In other cases they only occur on an attempt at volition, when the patient attempts to move or speak, when all control of the muscles seems to be lost. In such cases, if the patient can compose both mind and body, he may usually obtain some rest.

Notwithstanding the violence of these symptoms, they are scarcely ever attended by pains; and if perchance they are, it usually depends upon some complication, either functional or organic, and may be removed without in any way influencing the chorea.

This disease seldom attacks persons of a vigorous constitution, in adult life or in old age. The feeble of both sexes are liable to its attacks, although those between the ages of eight and sixteen are its most frequent subjects: it has also been known to attack young infants.

It more frequently attacks girls than boys, at the accession or approach of puberty; which may depend upon their more delicate conformation or effeminate habits, or perhaps their greater liability results from the retention of the catamenia, when the uterine organs are either unusually late or early in their development. This latter theory is rendered more probable by the fact that this disease usually subsides upon menstruation being fully established, and resists all treatment up to that period.

In the early stages of this disease, the gesticulations of the patient are often attended with remarkable contentment and good humor, and the absence of pain enables him to laugh with others at his own eccentricities.

As the disease advances, articulation is interfered with, so that the patient cannot be understood while talking; and deglutition is also sometimes so difficult that food enough cannot be swallowed to prevent great debility and emaciation. The mind may become impaired in its functions, accompanied by languor and vacuity of countenance, sometimes attended by a constant state of melancholy, and even in some cases, idiocy supervenes, the faculties retrograding to those of infancy.

In a few severe cases, the patient is not only unable to walk or to stand, but is also unable to sit upon a chair even, by reason of the convulsive movements of the limbs; but is compelled to assume the recumbent posture. In other cases, the patient can sit quietly, but upon rising and attempting to walk, a rotary motion of the body comes on, so that the patient turns rapidly round upon the toes, or is obliged to run swiftly about in a circle to keep from falling. In other cases the gesticulations are confined to one arm or one leg; in others, to one side of the body; and in others, again, to the face and head. In almost all cases, if the motions of one limb are restrained, they are transferred to another, or some other part of the body.

In some cases this disease has a semblance of being contagious. Numerous instances are on record of its being communicated by the force of example, or the habit of imitation; as in boarding schools, assemblies of religious fanatics, &c. One circumstance of this fact came under my own observation. In a large school where there were many girls, one, thirteen years of age, was attacked with symptoms of chorea, at first slight, but rapidly increasing upon her, until finally she had to be removed from school. Three other girls, about the same age as the first, were also successively attacked, that had been in the habit of imitating her while at play; two of the three became very intractable cases and were very difficult to cure, running several months. For this reason, the separation of choreic patients from other children should be insisted upon under all circumstances.

This disease may come on gradually or suddenly; in either case it may be idiopathic or symptomatic. It not infrequently supervenes upon an attack of remittent or intermittent fevers; in that case it may safely be considered symptomatic. It may be accompanied or preceded by impaired health, a disturbance of the digestive organs, constipation, worms, and other intestinal irritation.

It often commences at the period of the second dentition, when upon examination we find the old teeth remaining, the new ones appearing at their sides; in these cases if the old teeth are promptly extracted, if the patient is not entirely cured, we shall certainly find him much improved.

The predisposition to chorea is no doubt constitutional, and in many cases it would seem to be hereditary. It always attacks persons of a highly excitable, nervous, temperament, and usually at that period of life in which the nervous system is most excitable.

Its exciting causes are numerous; but they most frequently depend upon mental emotions, as fright, anger, religious fanaticism, disappointed love, over tasking the mind with study, &c., and it is never produced by physical causes, except those that are peculiarly operative upon the brain and nervous system. Such are puberty, anticipated or delayed, inflammation or irritation of the genital organs, suppressed discharges or eruptions, spinal irritation, long continued, from worms or disordered secretions; wounds or injuries about the head may also operate as an exciting cause.

The exact pathology of this disease has never been definitely settled, from the fact that its rare fatality affords so few opportunities for dissection. It has been known to occur in paroxysmal visitations from youth to advanced age without destroying life.

But there are many reasons pointing to the sensorium as the seat of the irritation upon which this disease depends; as its arising so often from mental causes, particularly an excited imagination; the injurious effects produced upon the mental faculties, often early in its progress, and almost certainly in very protracted cases; occasionally terminating in idiocy and insanity; the general absence of spasmodic action during sleep; also the fact that the involuntary muscles are seldom, if ever involved.

Fortunately it is of little practical importance to decide definitely in what particular part of the cerebral tissue the irritation is located, as it would not modify the therapeutic indications. Some suppose that the essence of this disease consists in a mobility of the nervous system, by which the control of the voluntary muscles is lost; others, in a morbid action of the faculty of volition. In either case, however, the same result is attained, and we are forced to the conclusion that the symptoms arise from an irregular excitement of some portion of the cerebral mass.

This disease is in most, if not in all cases, evidently associated with, if not dependent upon, an asthenic state of the system. The general history of the patients who suffer from chorea confirms the view that the nutrition of the brain, and indeed the whole nervous system, must be impaired. They are usually pale, feeble, persons, more or less infected with the matter of scrofula, or of rheumatism, or perhaps with some morbid matter peculiar to chorea itself, or generated by a depraved primary assimilation. And if we look at the muscles themselves, we will not find them in a healthy state, but on the contrary, we find the whole muscular system in a more or less soft and flabby condition; and if you compress the affected muscles, you do not find the firm resistance of a muscle contracting with its normal vigor, but rather that which indicates that the muscle contracts with feebleness, and is incapable of perfectly developing the muscular force.

I think no one can doubt, notwithstanding the localization of the phenomena of chorea, that it depends upon a depraved or poisoned state of the blood, to a great extent. This localization of chorea to one side, one limb, or to the muscles of the tongue, is by no means inconsistent with what we know of the laws governing morbid poisons: as for instance, the localization of the syphilitic poison upon the skin and periostium; the tonsils and lymphatic glands in the poison of scarlatina; of the lungs in measles; and the gastro-intestinal mucous membrane in cholera, &c.

If our views of the pathology of chorea are correct, the course of treatment is simple and plain; and yet I am aware that a great variety of treatment has been practiced. The older writers recommended bleeding and purging in all cases indiscriminately; this plan of treatment, however, has fallen deservedly into disrepute. It would be absurd to imagine that the ever changing forms that this disease assumes admit of the universal and indiscriminate application of any one remedy or specific; or that all cases may be successfully treated by any uniform plan. The age, sex, temperament, predisposing and exciting causes, period of the disease, as well as the previous treatment to which the patient may have been subjected its periodicity, and other modifying circumstances, must be taken into consideration in deciding upon the plan of treatment or the individual remedy.

It will be found, in a large majority of cases, that depletion will no be borne, and that an aggravation of symptoms will follow even active cathartics. The only exception to this rule is when the chorea depends upon some gastric irritation, or some gastric irritation coëxists with the chorea. But the tonic plan of treatment being confidently settled upon, it will be in point to notice the individual remedies of this class which are adapted to the treatment of chorea. There is scarcely a single vegetable or mineral tonic within the whole range of therapeutics that has not been used in the treatment of this disease. Cinchona or some of its preparations, of which quinia is preferred, is the main reliance with many. Others use iron, zinc, and the various mineral tonics; these various remedies often fail, although persevered in, and judicious regimen superadded; and still oftener the disease recurs after a short interval having only been suspended by these remedial agents.

I have learned to rely mainly upon the tonic powers of arsenic, in preference to any or all other remedies, and have never failed, I think, to effect a permanent and radical cure.

The arsenite of potassa, as existing in the formula of Fowler's solution, is the preferable mode of exhibition, and its dose should be graduated according to the ability of the stomach to receive it without nausea. Patients in general, varying from seven to sixteen years of age, may take at first from six to eight drops, morning and evening, and the dose should be gradually increased in quantity and frequency. Adults may take ten drops, increasing to fifteen, and even twenty, three times daily; and the use of it should be persevered in for a week or ten days after all spasmodic action of the muscles had disappeared. The only unpleasant effects are nausea or vomiting, if the dose be too large, and occasionally a tumefaction of the head and face if too long persisted in. I have used this remedy myself, and seen it used by others in their practice, in a large number of cases of chorea, without having witnessed any of the unpleasant results sometimes supposed to follow the use of arsenical preparations. My patients, some of them that I have had an opportunity of watching, have not only recovered from the chorea, but have since acquired healthy and vigorous constitutions.

But while dwelling upon the use of arsenic as a remedial agent in chorea I would not forget to add, that in most cases auxilliary treatment is necessary. I have never seen a case benefitted by active depletion, not even by drastic cathartics, although when constipation has been present, or the existence of worms has been suspected, I have interposed cathartics or anthelmintics, while at the same time persevering in the tonic treatment.

Nutritious diet, active exercise in the open air, and the cold bath, are useful adjuvants. When spinal irritation exists, counter irritation should be persevered in until the vertebral tenderness subsides.

The success of arsenic in this disease does not depend alone upon its tonic powers, in my opinion, as these powers exist to an equal if not a greater degree in other remedies, while in this disease they fail to produce the same effect.

Dr. Gregory and others maintain that arsenic acts by producing a strong impression upon the nervous system, other than simply a tonic effect, and refer as proof to the fact that intermittent and other paroxysmal diseases with and without periodicity are thus arrested.

The strong impression upon the nervous system made by arsenic interrupts that chain of actions in the body which have long been associated with convulsive movements of the limbs. These movements may be at first the result of some slight irritation in a portion of the sensorium, but afterward kept up by the power of habit.

I will subjoin two or three cases taken from my note-book:

*Feb.* 12, 1852. I was called to see a daughter of Mr. M., Franklin St. Her age was twelve years; she was very tall of her age, had grown very rapidly for the last two years; had never menstruated.

I found her suffering from a slight attack of chorea, confined to the muscles of the face, neck and left arm; she also had erysipelas of the left leg, extending from the knee nearly to the ankle. She had been treated about a year previous for chorea, by another physician of this city, who had given her various tonics, both vegetable and mineral, without relief, and finally resorted to arsenic, which relieved her in about four weeks.

I prescribed for her an emetic of ipecacuanha, followed by quinine and pulv. Doveri, at night, to induce sleep; also directed diluted alcohol as a local application to the leg.

After about five days, the erysipelas subsided, but the chorea increased so that the right arm and lower extremities became affected, the muscles of the tongue were also involved, so that she could scarcely utter a syllable intelligibly.

*Feb.* 24. I prescribed Fowler's solution in five drop doses, three times daily, and gradually increased the dose, until in fourteen days she took ten drops at a dose; this was continued fourteen days, when tumefaction under the eyes began to appear, and also an amelioration of the symptoms; the dose was reduced one-half, and continued for two weeks longer, when all the symptoms of chorea had disappeared. About this time, the catamenia appeared; since which she has enjoyed uniformly good health to the present date.

A daughter of Mr. I., of Perry, in this State, aged twelve years, called upon me in June, 1855. She had been suffering from chorea for about six months; had been treated by purgatives, antispasmodics, and different preparations of iron, without benefit; her bowels were much constipated, for which I gave her ol. terebinth. and ol. ricini, which removed a number of lumbricoid worms; after which, for a few days, the action of the muscles was less violent, but was soon as severe as at first. I then prescribed Fowler's solution, commencing with six drops, gradually increasing to ten, three times a-day.

This was continued for about two weeks, when she began to improve, and the dose was again reduced to seven drops, which was continued about six weeks, when convalescence was fully established.

*June* 9, 1857, I first saw Henry Q., Clinton St., aged 9 years. He had shown symptoms of chorea for about two weeks; he had also an attack of the same kind about a year previous, in the country. I can learn nothing definite of his treatment at that time.

When I saw him first, his disease was mostly confined to the left side; the muscles of the face and tongue, however, seemed more affected than those of the limbs. Upon examination, I found his teeth in a bad condition, several of the new teeth appearing by the side of the old ones; after extracting six of the old teeth, and relieving the constipation by mild cathartics, he was much better for a few days, but in about two weeks I found him worse than ever, when I prescribed Fowler's solution in six drop doses, three times a-day, gradually increased to ten, but this produced vomiting so that it was necessary to reduce the dose again to six drops, which was continued for about four weeks, when I found my patient entirely relieved.

Dr. Miner moved that the thanks of the association be tendered to the retiring president for the able and impartial manner in which he had performed the duties of his office for the past year. Which was seconded and carried.

Dr. Hutchins moved that the Association adjourn till the next Tuesday evening. Seconded and carried.

The Association then adjourned.

AUSTIN FLINT, Jr., M. D.,
Secretary.

